# Primary Diffuse Large B-Cell Lymphoma of the Urinary Tract: A Population-Based Analysis

**DOI:** 10.3389/fonc.2021.609882

**Published:** 2021-07-14

**Authors:** Zheng-Huan Liu, Lu-Chen Yang, Pan Song, Kun Fang, Jing Zhou, Zhu-Feng Peng, Qiang Dong

**Affiliations:** Department of Urology, Institute of Urology, West China Hospital, Sichuan University, Chengdu, China

**Keywords:** diffuse large B-cell lymphoma, urinary tract, SEER, prognosis, primary

## Abstract

**Objective:**

Diffuse large B-cell lymphoma (DLBCL) is the most common histopathological type of non-Hodgkin’s lymphoma, which may arise from various extranodal sites. Little is known about the clinical characteristics and survival outcomes of primary DLBCL of the urinary tract (UT). Thus, we conducted this study to explore the independent prognostic factors of patients with UT-DLBCL using the Surveillance, Epidemiology, and End Results (SEER) database.

**Materials and Methods:**

We searched the Surveillance, Epidemiology, and End Results (SEER) database for the data of patients diagnosed with UT-DLBCL between 1975 and 2016. Data, including demographic tumour stage and therapeutic strategies, such as surgical resection, radiation therapy, and chemotherapy, were collected. The impact of these factors on survival outcomes, including overall survival (OS) and disease-specific survival (DSS), was analysed using Kaplan–Meier curves.

**Results:**

Four-hundred and eighty-nine patients who met the inclusion criteria were enrolled in the data analysis. The median age was 69 years old. Most cases of UT-DLBCL (72.39%) originated from the kidney, followed by the urinary bladder (24.95%). Both surgical resection and chemotherapy can significantly improve OS and DSS. Patients older than 75 years had the worst survival outcomes. Stage IV DLBCL may be a poor prognostic factor.

**Conclusion:**

To the best of our knowledge, this is the largest population-based study of UT-DLBCL. Advanced age, male gender, lack of surgical resection or chemotherapy, and stage IV DLBCL were poor prognostic factors.

## Introduction

The most commonly diagnosed non-Hodgkin’s lymphoma (NHL) subtype is diffuse large B-cell lymphoma (DLBCL), which accounts for approximately 30% of NHL cases (1). Lymphomas can arise in almost every organ or site, and approximately one-third of the patients have extranodal origins (2). Among them, less than 5% were genito-urinary lymphomas (3). Furthermore, lymphomas of the UT are extremely rare; no more than 100 cases have been reported so far globally (4). The etiological exposure, clinical characteristics, and survival outcomes may vary for different sites of origin (5).

The use of recently established first-line therapy, including rituximab combined with cyclophosphamide, doxorubicin, vincristine, and prednisone (R-CHOP), has improved the survival outcomes of patients with DLBCL (6). However, questions about the treatment-related side effects, as well as long-term complications, remain unanswered. Increasing evidence has shown that extranodal sites demonstrate distinct clinical characteristics, survival outcomes, and require specific treatment (7, 8). Thus, studies on the clinical features and survival outcomes of DLBCL originating from the UT are limited.

This study explored the prognostic factors of patients with UT-DLBCL, based on data from the SEER database, to provide findings that will facilitate better clinic evaluations and more effective management of DLBCL.

## Materials and Methods

We utilized the SEER database to enrol eligible patients between 1975 and 2016, collected from 18 state registries in the USA. The patients diagnosed with lymphoma with the third edition of the International Classification of Diseases for Oncology (ICD-O-3) histology code of 9680 (DLBCL, not otherwise specified) in the database were included. Patients with DLBCL originating from sites other than the UT were excluded. In addition, all cases diagnosed before 1983 were excluded for the lack of the Ann-Arbor stage information. The patients without active follow-up and unknown Ann-Arbor stage, and those without histopathological confirmation of DLBCL, were also excluded from this study. Eligible patients were further identified based on the primary site, such as the kidney, ureter, urinary bladder, and urethra.

All the demographic, clinical, pathological, and survival data of the patients were extracted from the SEER database. The patients were divided into two groups based on the primary site: upper UT (UUT) group, with sites that include kidney, renal pelvis, and ureter; and lower UT (LUT) group, with sites that include urinary bladder and urethra. The treatments for patients with UT-DLBCL were also exported from the database, including radiotherapy, cancer-directed surgical resection, and chemotherapy. The ages of diagnosis were grouped into the following: 0–60, 60–75, and older than 75 years. The races were classified into white, black, and other. Importantly, the overall survival (OS) and disease-specific survival (DSS) were both analysed in this study.

The SEER database records survival as the number of months elapsed from the time of diagnosis of DLBCL to the day of death or the latest contact. For the estimation of disease-specific survival (DSS), only death attributable to the DLBCL was considered an exact event. To prevent confounding, we excluded patients diagnosed with more than one primary malignancy from the survival analysis.

### Ethical Approval

All data used in this study were from the public database, thus, ethical approval was required. We maintained the confidentiality of the data and reported the study results according to the SEER Research Data Use Agreement.

### Statistical Analysis

Continuous variables with normal distribution are presented as mean and standard deviation (SD); otherwise, median and inter-quartile range are considered. The differences in continuous variables were evaluated using the 2-sided analysis of variance (ANOVA) or the Mann-Whitney U-test according to their distribution. The categorical variables are presented as counts and percentages. Chi-squares or Fischer’s exact tests were applied to evaluate their differences. The survival curves were estimated using the Kaplan–Meier method and compared using the log-rank test. Cox proportional hazard models were used to identify the independent predictors of disease-specific mortality. Patients with missing data for one or more of the variables were excluded from the multivariable regression analysis. To allow adequate power for the Kaplan–Meier curves and the Cox model, the kidney cases were grouped with renal pelvis and ureter cases, whereas the urethral cases were grouped with the bladder cases. The statistical analysis was performed using IBM SPSS Statistics 24 software (IBM Corp., Armonk, NY, USA), and a p-value of less than 0.05 was considered statistically significant.

## Results

### Demographics and Clinical Features

Finally, we enrolled 489 eligible patients diagnosed with UT-DLBCL between 1975 and 2016 from the SEER database. The demographic and clinical characteristics of the patients are summarized in **Table 1**. The mean age at diagnosis was 69 years. There were 143 (29.24), 166 (33.95%), and 180 (36.81%) patients in the < 60, 60–75, and ≥ 75 years age-at-diagnosis groups, respectively. The gender distributions were not different. Most of the patients were white (84.04%); 15.54% were black or other. The most common primary site was the kidney (72.39%), followed by the urinary bladder (UB) (24.95%).

**Table 1:** Characteristics of patients with DLBCL (N=489).

	Number of cases (%)
Age at diagnosis (range)	69 (4-97)
<60	143 (29.24)
60-75	166 (33.95)
≥75	180 (36.81)
Gender	
Female	221 (45.19)
Male	268 (54.81)
Marriage status	
Married	259 (52.97)
Single	211 (43.15)
Unknown	19 (3.88)
Race	
White	411 (84.04)
Black	38 (7.77)
Other	38 (7.77)
Unknown	2 (0.42)
Primary site	
Kideny	354 (72.39)
Renal Pelvis	3 (0.61)
Ureter	4 (0.82)
Urinary Bladder	122 (24.95)
Urethral	6 (1.23)
Surgery	
Yes	213 (43.56)
No	276 (56.44)
Radiation	
Yes	38 (7.77)
No	451 (92.23)
Chemotherapy	
Yes	356 (72.80)
No	133 (27.20)
Ann Arbor Stage	
Stage I	167 (34.15)
Stage II	119 (24.34)
Stage III	35 (7.16)
Stage IV	168 (34.35)

DLBCL, Diffuse large B cell lymphoma.

Surprisingly, cancer-directed surgery was performed in about half of the cases (56.44%). Forty-one patients (7.77%) underwent radiation therapy. The majority of these cases (72.80%) underwent chemotherapy. The most common Ann-Arbor stage at presentation was stage I (34.15%), followed by stage IV (34.35%).

### Prognostic Factors of Survival Outcomes

**Figure 1:**
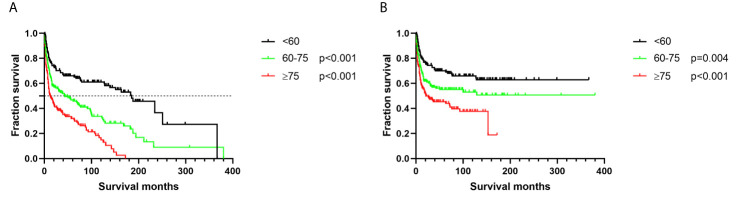
Survival analysis based on age: **(A)** overall survival; **(B)** disease-specific survival.

Patients aged ≥ 75 years had the worst survival outcomes, with OS of only 8 months and DSS of 12 months (**Figure 1** and **Table 2**, p < 0.001). The 5-year OS for the < 60 and 60–75 years age-at-diagnosis groups of patients were 64.29% and 43.51%, respectively; the 5-year OS was 27.10% for the ≥ 75 years group. The 5-year DSS for these three groups were 69.06%, 51.36%, and 39.23%, respectively.

**Table 2:** Univariate survival analysis.

	OS	HR (95% CI)	*p-value*	DSS	HR (95% CI)	*p-value*
Age						
<60	185	Reference		Not reached	Reference	
60-75	31	2.080 (1.529-2.830)	<0.001*	100	1.703 (1.187-2.443)	0.004*
≥75	8	3.564 (2.634-4.822)	<0.001*	12	2.671 (1.893-3.770)	<0.001*
Gender						
Female	43	Reference		Not reached	Reference	
Male	17	1.226 (0.985-1.527)	0.069	35	1.282 (0.986-1.666)	0.063
Marital status						
Married	30	Reference		71	Reference	
Single	22	1.068 (0.854-1.335)	0.566	100	1.011 (0.776-1.316)	0.937
Race						
White	27	Reference		76	Reference	
Black	25	0.926 (0.614-1.397)	0.715	100	0.999 (0.616-1.620)	0.998
Other	17	1.128 (0.754-1.687)	0.558	72	1.188 (0.750-1.883)	0.463
Primary site						
UUT	26	Reference		67	Reference	
LUT	27	1.080 (0.848-1.376)	0.533	128	0.892 (0.660-1.205)	0.455
Surgery						
Yes	63	Reference		Not reached	Reference	
No	16	1.310 (1.051-1.633)	0.016*	21	1.624 (1.241-2.127)	<0.001*
Radiation						
Yes	87	Reference		Not reached	Reference	
No	21	1.419 (0.936-2.152)	0.099	63	1.995 (1.116-3.568)	0.020*
Chemotherapy						
Yes	57	Reference		153	Reference	
No	3	1.998 (1.583-2.522)	<0.001*	4	2.241 (1.708-2.940)	<0.001*
Ann Arbor Stage						
Stage I	57	Reference		Not reached	Reference	
Stage II	77	0.927 (0.684-1.257)	0.625	Not reached	1.004 (0.691-1.460)	0.982
Stage III	21	1.261 (0.815-1.951)	0.297	24	1.439 (0.873-2.370)	0.153
Stage IV	10	1.537 (1.188-1.987)	0.001*	13	1.827 (1.340-2.489)	<0.001*

OS, Overall survival; DSS, disease specific survival; HR, Hazard ratio; CI, Confidence interval; UUT, Upper Urinary Tract; LUT, Lower Urinary Tract; *p < 0.05.

**Figure 2:**
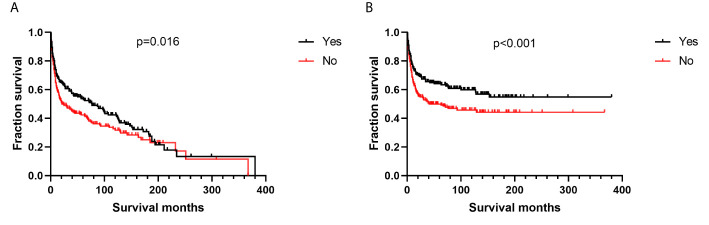
Survival analysis based on surgical resection: **(A)** overall survival; **(B)** disease-specific survival.

**Figure 3:**
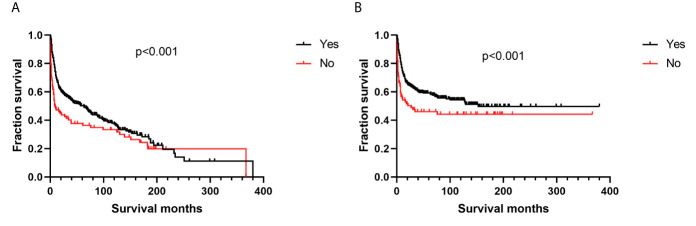
Survival analysis based on chemotherapy: **(A)** overall survival; **(B)** disease-specific survival.

**Figure 4:**
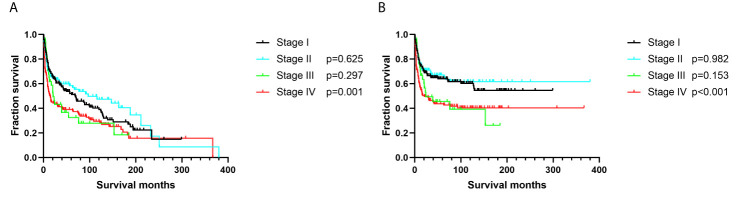
Survival analysis based on Ann-Arbor stage: **(A)** overall survival; **(B)** disease-specific survival.

Additionally, we found no differences in OS or DSS in the gender and race groups (**Table 2**). There was also no difference between the survival outcomes of the UUT and LUT groups (**Table 2**). Regarding the treatment strategies, patients who had undergone cancer-directed surgery had significantly longer OS than those who had not (**Figure 2A** and **Table 2**, 63 *vs.* 16 months, HR: 1.310, 95%CI: 1.051–1.633, p=0.016). The same outcome was found for DSS in the two groups (**Figure 2B** and **Table 2**, HR: 1.624, 95%CI: 1.241–2.127, p < 0.001), whereas radiotherapy had no survival benefit to patients with UT-DLBCL. Not surprisingly, patients who were not undergoing chemotherapy had approximately twice the risk of death as those who were (**Figure 3** and **Table 2**, p < 0.001). Finally, we found that patients with Stage IV DLBCL had worse survival Kaplan-Meier curves than the others (**Figure 4** and **Table 2**).

### Multivariate Survival Analysis

The outcomes of the multivariate survival analysis are summarized in **Table 3**. Variables, including age, gender, primary site, treatment strategies, and Ann-Arbor stage, were analysed. Older patients had worse OS and DSS than younger patients. Different from the above results, males had worse OS (HR: 1.336, 95% CI: 1.061–1.681, p=0.014) and DSS (HR: 1.340, 95%CI: 1.020–1.759, p=0.036). Patients who had undergone surgical resection had better OS and DSS than those who had not (p < 0.05). In addition, chemotherapy had a remarkable effect on survival in UT-DLBCL (p < 0.001). Patients with stage IV UT-DLBCL tended to have worse survival than those with Stage I UT-DLBCL; this was not only for OS (HR:1.606, 95%CI: 1.202–2.146, p=0.001) but also for DSS (HR:1.835, 95%CI: 1.298–2.594, p=0.001). The other variables did not affect the survival outcomes of UT-DLBCL.

**Table 3:** Multivariate survival analysis.

	OS		DSS	
	HR (95% CI)	*p-value*	HR (95% CI)	*p-value*
Age				
<60	Reference		Reference	
60-75	2.253 (1.646-3.086)	<0.001*	1.815 (1.260-2.615)	0.001*
≥75	3.592 (2.639-4.890)	<0.001*	2.645 (1.863-3.755)	<0.001*
Gender				
Female	Reference		Reference	
Male	1.336 (1.061-1.681)	0.014*	1.340 (1.020-1.759)	0.036*
Primary Site				
UUT	Reference		Reference	
LUT	1.248 (0.940-1.657)	0.125	1.183 (0.837-1.671)	0.341
Surgery				
Yes	Reference		Reference	
No	1.301 (1.014-1.670)	0.039*	1.539 (1.136-2.084)	0.005*
Radiation				
Yes	Reference		Reference	
No	1.315 (0.829-2.087)	0.245	1.521 (0.810-2.858)	0.192
Chemotherapy				
Yes	Reference		Reference	
No	2.031 (1.595-2.585)	<0.001*	2.405 (1.813-3.191)	<0.001*
Ann Arbor Stage				
Stage I	Reference		Reference	
Stage II	1.013 (0.736-1.395)	0.938	1.069 (0.723-1.580)	0.738
Stage III	1.113 (0.714-1.736)	0.636	1.325 (0.795-2.206)	0.280
Stage IV	1.606 (1.202-2.146)	0.001*	1.835 (1.298-2.594)	0.001*

OS, Overall survival; DSS, disease specific survival; HR, Hazard ratio; CI, Confidence interval; UUT, Upper Urinary Tract; LUT, Lower Urinary Tract; *p < 0.05.

### Subgroup Analysis of Kidney Versus UB

**Figure 5:**
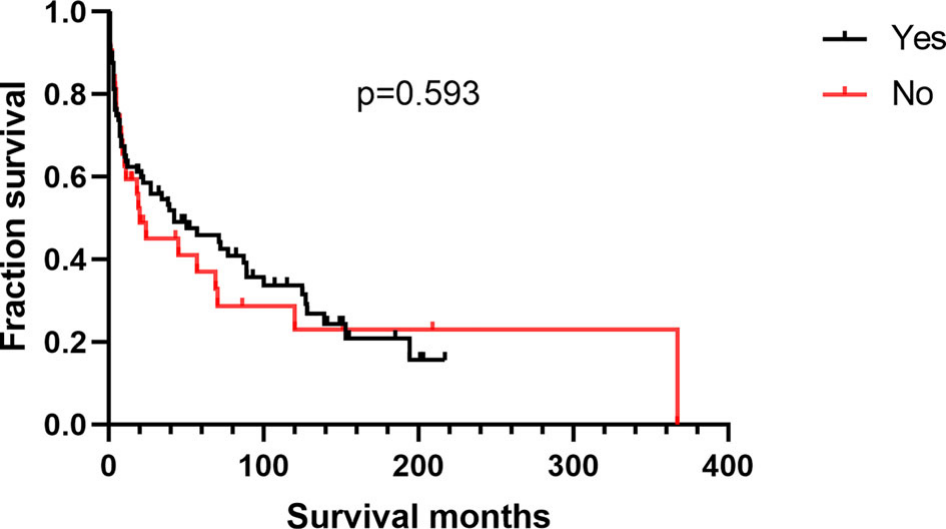
Overall survival analysis based on surgical resection for urinary bladder DLBCL.

**Figure 6:**
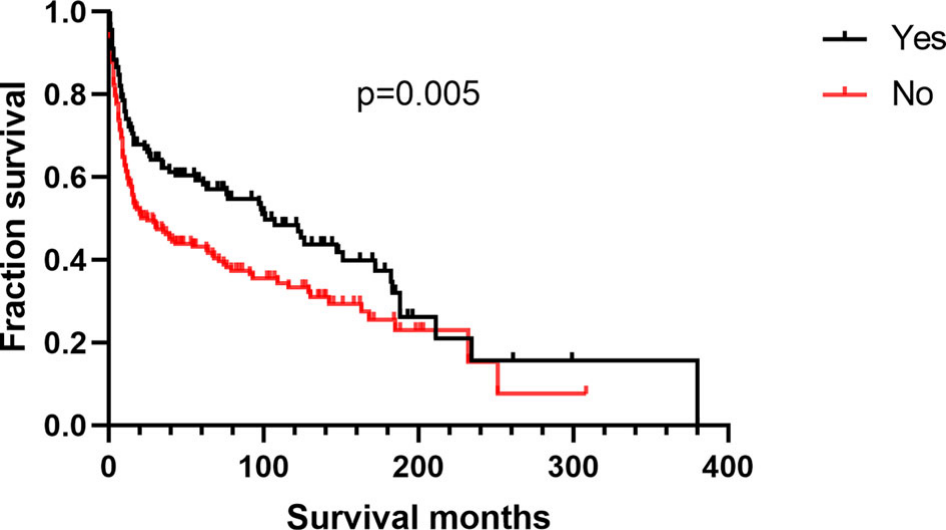
Overall survival analysis based on surgical resection for kidney DLBCL.

Patients with kidney DLBCL were younger than those with UB-DLBCL (**Table 4**, p < 0.001). Kidney DLBCL was predominant in males (59.89%), and UB-DLBCL was predominant in females (59.84%). More than half of the cases of UB-DLBCL (56.56%) were diagnosed as Stage I, compared with only 25.71% of the cases of kidney DLBCL (p < 0.001). Interestingly, for patients with DLBCL of the UB, cancer-directed surgery had no survival benefit (**Figure 5**, p=0.593), which was different from those with kidney DLBCL (**Figure 6**, p=0.005).

**Table 4:** Subgroup analysis of patients with kidney versus UB DLBCL.

Characteristics		Kidney	UB	p-value
Age, years	Median	68	76	<0.001a*
Gender, n	Female	142	73	<0.001b*
	Male	212	49	
Ann-Arbor stage, n	I	91	69	<0.001b*
	II	88	26	
	III	26	9	
	IV	149	18	
Surgery, n	Yes	119	87	<0.001b*
	No	235	35	

UB, Urinary Bladder; DLBCL, Diffuse large B cell lymphoma; *p < 0.05

(a)Analysis of variance (ANOVA).

(b)Chi-square test.

## Discussion

Lymphomas can arise from any site, and extranodal lymphomas account for one-third of all cases (2). DLBCL is the most common subtype of NHL. However, lymphomas arising from the UT account for less than 5% of the extranodal NHL (3). For instance, less than 100 cases of UT lymphomas have been reported so far (4). Thus, UT-DLBCL is an extremely rare NHL. As far as we know, this is the first study to explore the clinical features and survival outcomes for UT-DLBCL in a relatively large population, and this may help urologists manage UT-DLBCL better.

In this study, we found that patients aged ≥ 60 years were more likely to suffer from UT-DLBCL. In previous studies, a multistep carcinogenic model of solid tumours (9) showed that the incidence of UT-DLBCL increases exponentially with aging. Two reasons may contribute to this phenomenon: first, the incidence of chronic inflammation can gradually increase with aging, which is one of the major risk factors of mortality (10). Secondly, the immune function of older patients may decline. The deregulation of the immune system is characterised by thymic involution, which leads to a gradual decline in the development of naïve T cells (11). Moreover, several genes or molecules change with aging, and this may promote the development of DLBCL (12).

Importantly, we found that age was an independent prognostic factor of OS and DSS. The morality of patients aged ≥ 75 years was 2–3 times higher than that of younger patients. A previous study demonstrated that older age is a poor prognostic factor of UT lymphomas (13).

Additionally, we found that the female gender was also an independent prognostic factor, which is consistent with the theory that female patients with B-cell lymphomas may have a better response to chemotherapy combined with rituximab (14, 15).

Furthermore, we found that surgical resection may be beneficial for patients with UT-DLBCL, especially for those with kidney DLBCL. However, there was no beneficial effect of surgery on patients with UB-DLBCL. There are no surgical recommendations for UB lymphomas, mainly because of the morbidity and mortality associated with surgery (16). Regarding radiotherapy, our results suggested that radiation treatment had no benefit on UT-DLBCL patients. Notably, this result may be biased because there are inherent limitations of SEER radiotherapy data. Radiation therapy is an important treatment for nodal DLBCL, and the International Lymphoma Radiotherapy Group includes in its guidelines that “pelvic lymphomas involve the bladder or gynaecological organs” and recommends the use of radiation therapy. However, no specific indications have been outlined for kidney lymphomas (17). Thus, the effect of radiation therapy needs to be further explored in more targeted research.

Chemotherapy, as the most used treatment in this study population, can significantly improve prognosis. Chemotherapy is still the mainstay of nodal DLBCL treatment (18). However, a previous study showed that the primary site of extranodal NHL determines the effectiveness of chemotherapy; they found no significant improvement in patients with genitourinary DLBCL who had undergone chemotherapy with rituximab (19). The dissension may be attributed to the confounding of the surgery, as there are no data on the use of combination with other therapy. Thus, further studies are urgently needed to clarify the survival benefits of chemotherapy in patients with UT-DLBCL.

There were several differences between the UB and kidney DLBCL groups. Similar to the previous results, patients with UB-DLBCL were predominantly female, whereas those with kidney DLBCL were predominantly male (13). More than half of the cases of UB-DLBCL (56.56%) were diagnosed at Stage I, compared with only 25.71% of the cases of kidney DLBCL. The chief complaints in patients with UB-DLBCL were mainly haematuria, and this may have facilitated early diagnosis (20). More patients with UB-DLBCL than those with DLBCL underwent surgical resection. Patients with UB-DLBCL have more access to excisional biopsy than those with kidney tumours.

This study has several limitations, including a lack of data on the treatments, especially chemotherapy and combined therapy. Thus, future research involving a larger population is needed to confirm the findings.

## Conclusion

This is the first study to demonstrate the effect of demographic and clinical features of patients with UT-DLBCL on outcome prediction. Advanced age, male gender, and Stage IV were all important poor prognostic factors for primary UT-DLBCL. Surgical resection may be beneficial for UT-DLBCL. Chemotherapy was the mainstay of treatment of patients with UT-DLBCL.

## Data Availability Statement

Publicly available datasets were analyzed in this study. This data can be found here: Surveillance, Epidemiology, and End Results (SEER) database (https://seer.cancer.gov/).

## Author Contributions

Z-HL, L-CY and JZ conceived and designed the study, collected and analysed the data, and wrote the manuscript. PS and KF analysed the data. Z-FP and QD reviewed and edited the manuscript. All authors contributed to the article and approved the submitted version.

## Funding

The work is supported by the 1.3.5 project for disciplines of excellence, West China Hospital, Sichuan University (ZY2016104) and the National Natural Science Fund of China (81771569).

## Conflict of Interest

The authors declare that the research was conducted in the absence of any commercial or financial relationships that could be construed as a potential conflict of interest.
